# Longitudinal associations of academic stress with eating related patterns, nutrition, somatic indicators, and depressive symptoms in university students: A study protocol

**DOI:** 10.1371/journal.pone.0352779

**Published:** 2026-07-07

**Authors:** Pía Rojas-Cárdenas, Gabriela Nazar, Juan Luis Castillo-Navarrete

**Affiliations:** 1 Programa de Doctorado en Salud Mental, Facultad de Medicina, Universidad de Concepción, Concepción, Chile; 2 Carrera de Nutrición y Dietética, Facultad de Ciencias de la Salud, Universidad Adventista de Chile, Ñuble, Chile; 3 Departamento de Psicología, Facultad de Ciencias Sociales, Universidad de Concepción, Concepción, Chile; 4 Departamento de Tecnología Médica, Facultad de Medicina, Universidad de Concepción, Concepción, Chile; 5 Programa de Neurociencia, Psiquiatría y Salud Mental, NEPSAM, Universidad de Concepción, Concepción, Chile; Methodist University Cape Fear Valley Health School of Medicine, UNITED STATES OF AMERICA

## Abstract

**Introduction:**

Academic stress (AS) can be understood as a multidimensional form of chronic stress that has been linked to adverse patterns across physical and mental health in university students. This protocol outlines a longitudinal, observational study to examine within-semester associations between AS and indicators of nutritional status, eating-related patterns, sleep quality, and depressive symptoms. By integrating physiological, behavioral, and psychological indicators within a repeated-measures framework, the study aims to generate evidence that can inform prevention-focused actions and early support strategies in university health contexts.

**Hypotheses:**

(i) Higher AS will be longitudinally associated with less favorable eating-related patterns, nutritional and somatic indicators, and higher depressive symptoms in university students. (ii) Higher AS will be associated with altered post-awakening cortisol dynamics, specifically a lower cortisol awakening response (CAR) and lower AUCi, together with a higher AUCg, and with higher salivary alpha-amylase (sAA) levels, consistent with stress-related activation of HPA-axis and sympathetic pathways.

**Design and participants:**

A longitudinal, observational, nonexperimental design with repeated measurements will be implemented across three assessment cycles during an academic semester. A feasibility-based convenience sample will be recruited from undergraduate students (2nd to 4th year) enrolled in the Faculty of Medicine, University of Concepción (Chile). Students receiving psychological or pharmacological treatment will be eligible to reflect real-world heterogeneity and support ecological validity.

**Methods:**

Data will be collected through standardized questionnaires, nutritional assessments, biological sampling, and wearable-derived somatic indicators. Electronic surveys administered via REDCap will assess AS, perceived stress, eating-related patterns, and depressive symptoms. Diet will be assessed through interviewer-administered nutritional interviews, including repeated 24-hour dietary recalls treated as time-specific observations and modeled longitudinally as time-varying measures, and complemented by diet-quality and dietary inflammatory potential indices. Wearable devices will record nonclinical somatic indicators, including heart rate, oxygen saturation, and sleep-related metrics during monitoring periods. Saliva will be collected twice per week during each assessment cycle to quantify salivary cortisol dynamics and sAA activity, and peripheral blood samples obtained at baseline and end of semester will be used to determine lipid profile, fasting glucose, albumin, globulin, and total proteins.

**Statistical analysis:**

Analyses will include descriptive and bivariate summaries, followed by multivariable models appropriate to outcome type. Longitudinal associations will be examined using mixed-effects models, and temporal cross-lagged associations will be explored using random-intercept cross-lagged panel models across the three assessment cycles. All inferences will be framed as associational given the observational design.

**Expected results:**

Rather than prespecifying outcomes, this protocol is expected to generate longitudinal evidence on how within-semester variation in AS aligns with eating-related patterns, diet, nutritional and somatic indicators, depressive symptoms, and stress-related physiological indicators (salivary cortisol dynamics and sAA activity). The integrated, feasibility-oriented measurement framework may serve as a replicable template for future research and inform prevention-focused actions to support student well-being in university settings.

## Introduction

Stress is a major public health concern with consequences for both physical and psychological well-being [1]. In university settings, this issue is particularly relevant because students are commonly exposed to concurrent academic and social demands that may exceed their coping resources [2].

Evidence in student populations indicates that higher stress levels are associated with depressive symptoms [3], poorer sleep and related behavioral alterations [4,5], and stress-related changes in eating behavior [6] together with dysregulation of stress-responsive systems such as the hypothalamic-pituitary-adrenal (HPA) axis and the autonomic nervous system (ANS) [7,8]. In this context, this protocol describes the methodological design of a longitudinal study aimed at examining the association between academic stress (AS) and nutritional, somatic, and psychological indicators among university students, providing an empirical basis to inform prevention and early intervention efforts.

### Physiological mechanisms associated with stress

From a physiological standpoint, stress can be understood as an adaptive response to environmental or internal stressors that engages neuroendocrine, immune, and behavioral systems [7]. This response is mainly coordinated through the hypothalamic-pituitary-adrenal (HPA) axis and the autonomic nervous system (ANS) [8,9]. When a stressor is perceived, the HPA axis initiates the release of corticotropin-releasing hormone (CRH) by the hypothalamus, followed by adrenocorticotropic hormone (ACTH) secretion by the anterior pituitary, ultimately stimulating glucocorticoid synthesis, predominantly cortisol, in the adrenal cortex [9,10]. Cortisol contributes to energy mobilization and the regulation of immune and cardiovascular processes, and salivary cortisol is widely used as a noninvasive indicator of HPA-related activity in stress research [11,12]. In parallel, sympathetic activation promotes catecholamine release, leading to acute cardiovascular changes and increases in salivary alpha-amylase (sAA), an enzyme commonly used as a noninvasive indicator of sympathetic arousal in behavioral and psychophysiological studies [13–15].

Although acute stress responses are adaptive, sustained activation can lead to maladaptive changes that compromise well-being [8]. Chronic stress exposure has been associated with dysregulated cortisol output, sleep disturbances, immunological alterations, increased cardiovascular risk, and greater vulnerability to affective disorders, including major depressive disorder [9,11,12]. At the neurobiological level, stress-related alterations have been described in the hippocampus and broader limbic circuits involved in emotional and cognitive regulation [16]. In academic settings, these pathways may translate into concomitant disturbances in eating behavior, sleep, and mood, supporting the rationale for an integrated assessment of psychological, behavioral, and physiological indicators in university students.

This study will use salivary cortisol and sAA as noninvasive indicators of HPA axis and ANS (sympathetic) activity, respectively, which have been widely applied to characterize stress-related activation in nonclinical and student populations [17–19]. For cortisol, we will focus on the cortisol awakening response (CAR), capturing the rapid post-awakening increase, and we will compute the most commonly used derived indices from serial post-awakening measurements, including the area under the curve with respect to increase (AUCi) and with respect to ground (AUCg), which allow estimation of reactivity and overall output within the sampling window [20].

However, the available evidence still shows important limitations, including the predominance of cross-sectional designs, the limited integration of physiological, behavioral, and psychological indicators within a single analytical framework, and the relative scarcity of studies specifically focused on academic stress as an operationalized construct [7,21]. These gaps support the need for longitudinal protocols capable of examining temporal relationships using an integrative approach.

### Academic stress

AS can be conceptualized as a transactional phenomenon that emerges when academic demands are appraised as taxing or exceeding a student’s perceived coping resources [21,22]. This construct is common in university settings and has been linked to adverse consequences for health, academic functioning, and overall well-being. However, its boundaries have often been blurred by the coexistence of related terms such as “student stress,” “burnout,” and “academic anxiety,” which complicates conceptual differentiation and contributes to inconsistent operationalization across studies [23]. Consistent with this heterogeneity, the available instruments vary widely and frequently adopt unidimensional or decontextualized approaches that capture diverse sources of stress without embedding them within a systemic, integrative framework [24–27].

To address this conceptual and methodological diversity, this protocol adopts the systemic cognitivist model proposed by Barraza-Macías, which integrates general systems theory with transactional stress theory [23,28]. Within this framework, AS is represented through three interrelated components: academic stressors, indicators of systemic imbalance, and coping strategies. The model proposes that students face specific educational demands (input) that may trigger an imbalance reaction, followed by coping efforts aimed at restoring homeostasis (output). The SISCO Academic Stress Inventory was developed to operationalize these components. In Chile, both the original instrument and its expanded version (SISCO-II-AS) have been validated in university samples, showing robust psychometric properties [29,30]. The SISCO-II-AS retains the original subscales, refines symptom assessment by differentiating physical, psychological, and behavioral reactions, and incorporates reference norms to support standardized interpretation [30].

A clear and theoretically grounded operational definition of AS is essential to distinguish it from related constructs, reduce interpretive ambiguity, and support transparent sample delimitation [21]. Accordingly, this protocol uses the systemic cognitivist model and the SISCO-II-AS inventory as its conceptual and methodological foundation, given their contextual relevance and empirical support in Chilean university populations [23,29,30]. This approach enables AS to be examined as a multidimensional phenomenon plausibly linked to behavioral and somatic indicators relevant to student health, including eating-related patterns, sleep, cardiovascular risk indicators, and depressive symptoms.

### Impact of stress on eating, nutritional, somatic, and psychological variables

Stress is a key determinant of both physical and mental health. Its influence may occur through sustained activation of stress-responsive systems, including the HPA axis and the ANS, and through indirect pathways involving stress-related changes in daily functioning. In student and general populations, these effects have been linked to disruptions in eating-related behaviors, diet quality, sleep patterns, emotional well-being, and broader health outcomes [6,7].

### Stress and eating behavior

Chronic stress engages the HPA axis and the sympathetic branch of the ANS and has been linked to changes in appetite-related signaling, including cortisol, ghrelin, and leptin dynamics [31–33]. Sustained cortisol exposure may contribute to a shift toward highly palatable, energy-dense foods, often framed as stress-related food reward, through interactions between metabolic signals and neural reward processes [34–36]. These effects are plausibly supported by reward-circuit mechanisms, including pathways involving the nucleus accumbens, which may heighten motivation for palatable foods under stressful conditions [6,37].

In university settings, stress-related eating changes are particularly relevant because many students begin managing meals more independently, and patterns established during this period may persist into adulthood [38,39]. During high academic demand, increases in fast food, snacks, and high-fat or high-sugar intake have been reported, alongside lower consumption of fruits and vegetables [40–42]. Stress responses in eating can vary, presenting as higher or lower intake depending on individual factors and situational characteristics [40]. A commonly described pattern is repetitive, unplanned intake of small amounts of food, frequently termed grazing, which may function as a short-term coping behavior during exam periods but has been linked to less adaptive eating patterns over time [43,44].

Prolonged stress exposure has also been associated with heightened motivation for ultra-processed foods, and some authors describe an addictive-type pattern of consumption characterized by loss of control, although this does not constitute a clinical diagnosis [45–48]. Despite these associations, most studies remain cross-sectional, seldom focus specifically on academic stress as an operationalized construct, and rarely incorporate physiological indicators within an integrated model, limiting inference about temporal ordering and mechanisms [6,49].

### Stress and diet

Diet is another domain that may be shaped by stress-related changes in daily functioning. Population evidence highlights that healthier eating patterns are associated with better mental health and health-related quality of life [50]. In young adults, higher perceived stress has also been linked to poorer diet quality and greater consumption of fast foods and sugar-rich products, often accompanied by higher intake of stimulants such as coffee and energy drinks [40].

A key pathway connecting diet to health is inflammation. Dietary patterns characterized by higher intake of ultra-processed foods and energy-dense products have been associated with a more proinflammatory profile, and proinflammatory dietary patterns have been linked to depressive symptoms and related mental health outcomes in observational studies [51,52]. In university samples, diet-related inflammatory potential has also been examined in relation to stress and anxiety [53,54]. Beyond mental health, higher proinflammatory dietary scores have been associated with less favorable cardiometabolic and endocrine biomarker profiles in large datasets, although much of the evidence remains cross-sectional [55,56].

Stress may also affect nutritional status through sustained glucocorticoid exposure. Higher cortisol output has been associated with adverse body composition features and altered autonomic balance in young adults, and mechanistic evidence supports a role for glucocorticoid signaling in adipose-related pathways [57–59]. Together, these findings support an integrative approach to AS during university life, incorporating dietary patterns and nutritional status alongside physiological and psychological indicators.

### Stress and somatic variables: Sleep and cardiovascular risk factors

#### Stress and sleep.

Sleep is a regulated physiological process, and sleep quality is commonly described using core features such as sleep latency, nocturnal awakenings, wakefulness after sleep onset, and sleep efficiency [60]. Adequate sleep is a recognized determinant of physical and mental health and of day-to-day functioning. Sleep and stress are closely interrelated through circadian regulation and stress-responsive systems. Cortisol secretion follows a diurnal rhythm and contributes to the organization of sleep–wake cycles and adaptive responses to environmental demands [9]. Accordingly, evidence supports a bidirectional association: higher stress can impair sleep quantity and quality, and persistent sleep difficulties can contribute to physiological and emotional vulnerability that may, in turn, intensify stress-related activation [61,62].

During university years, sleep is frequently compromised. Studies in student populations report reduced sleep duration and poorer sleep quality, particularly during demanding academic periods [63,64]. Sleep restriction has also been associated with downstream disturbances relevant to this protocol, including changes in eating behavior and dietary intake patterns, alongside irritability and reduced cognitive control that may heighten perceived stress [4,5,65,66].

#### Stress and cardiovascular risk factors in young adults.

Stress contributes to cardiovascular risk through neuroendocrine and autonomic pathways that influence hemodynamic and metabolic regulation. Chronic activation of the HPA axis and the ANS can increase cortisol and catecholamine output, with downstream effects on heart rate and blood pressure [67]. In academic contexts, exposure to sustained demands such as examinations and performance pressure has been linked to transient increases in cardiovascular parameters and stress-related physiological reactivity [68,69]. Although these responses are often reversible in the short term, their repetition over time may contribute to risk-relevant processes, including autonomic imbalance and vascular dysfunction [67,70].

In young adults, clinically established cardiovascular disease remains uncommon, yet behavioral risk factors are highly prevalent, including unhealthy diet, alcohol use, smoking, sleep problems, and physical inactivity [71]. These risk factors can cluster and interact with stress-related physiology, supporting early assessment strategies in university populations. In this context, lipid and metabolic indicators are frequently used to characterize cardiometabolic risk in younger individuals and to support prevention-focused approaches [72].

#### Stress and depressive symptoms.

Stress plays a central role in the onset and maintenance of depressive symptoms through interacting biological and psychological pathways. Prolonged stress exposure can disrupt neuroendocrine regulation and affective processing, increasing vulnerability to persistent low mood and reduced adaptive capacity [10,73].

One key mechanism involves sustained HPA axis dysregulation, which may contribute to prolonged cortisol output and downstream alterations in neurotransmitter systems implicated in mood regulation, including serotonin, norepinephrine, and dopamine [35,74]. These changes may compromise neuroplasticity in stress-sensitive regions such as the hippocampus and promote proinflammatory signaling, reinforcing a feedback loop that sustains depressive symptomatology [10,73,75]. In parallel, chronic stress can alter reward-related dopaminergic function within the nucleus accumbens, which may contribute to anhedonia and reduced motivation, core features of depression [76,77].

### Individual variability in stress response

Stress does not affect individuals uniformly. Interindividual variability in physiological and behavioral stress reactivity reflects differences in both response magnitude and temporal patterning, and individual characteristics such as coping resources, dietary restraint, and biological sex may shape stress-related changes in eating behavior [6,40].

#### Summary and rationale.

Across the preceding sections, AS has been framed as a multidimensional phenomenon with potential implications for several domains of student health. The evidence reviewed supports plausible links between stress exposure and eating-related patterns, diet quality, nutritional status, sleep, cardiometabolic risk indicators, and depressive symptoms through interacting physiological, psychological, and behavioral pathways [7,67].

However, most available studies remain limited by cross-sectional designs and by the frequent use of general stress measures rather than constructs explicitly operationalized as AS, which restricts understanding of temporal relationships and within-person dynamics across the academic semester [6,21]. These limitations justify longitudinal protocols that integrate behavioral, physiological, and psychological indicators within a unified analytical framework.

Accordingly, this protocol addresses these gaps using a multidimensional, longitudinal approach focused on AS as an explicitly operationalized construct, assessed with the SISCO-II-AS framework [29,30]. We integrate physiological indicators (salivary cortisol and sAA), eating-related patterns (including snacking-related behavior and addictive-type UPF consumption), nutritional indicators (diet and nutritional status), somatic indicators (sleep and cardiometabolic risk factors), and depressive symptoms to capture stress responses across an academic semester. The originality of this study lies in combining repeated measurements with an integrative analytic strategy, overcoming key limitations of the predominantly cross-sectional literature in this field [6,21].

#### Problem and research question.

Mental health problems and cardiometabolic risk factors represent a substantial public health burden, and university students exposed to higher levels of AS may be particularly vulnerable. However, much of the available evidence remains cross-sectional, relies on general stress measures rather than AS as an explicitly operationalized construct, and rarely integrates physiological indicators with behavioral and psychological outcomes within a single longitudinal framework [6,21]. To address these gaps, this protocol applies a repeated-measures approach across an academic semester, operationalizing AS using the systemic cognitivist model through the SISCO-II-AS framework and integrating salivary cortisol and sAA indicators with nutritional, somatic, and depressive symptom measures [29,30]. The guiding research question is: *What is the longitudinal association between academic stress and eating-related patterns, diet and nutritional status, sleep and cardiometabolic indicators, and depressive symptoms across an academic semester in university students?*

## Method

Given that the research question focuses on examining longitudinal associations between academic stress (AS) and nutritional status, cardiometabolic risk indicators, and depressive symptoms, as well as their relationships with eating-related patterns, sleep quality, and diet across an academic semester, and considering the limited availability of longitudinal studies integrating physiological and behavioral indicators in university contexts [6,21], we propose the following hypotheses: (i) AS is longitudinally associated with eating-related patterns, nutritional and somatic indicators, and depressive symptoms in university students; and (ii) AS is associated with variation in post-awakening cortisol dynamics, reflected in CAR, AUCi, and AUCg within the sampling window as indicators of HPA-axis related activity, as well as with variation in salivary alpha-amylase (sAA) levels as an indicator of sympathetic arousal.

**General objective:** To examine the longitudinal associations between AS and indicators of nutritional status, eating-related patterns, sleep quality, and depressive symptoms across an academic semester among university students.

**Specific objectives:** (i) To characterize physiological correlates of AS by assessing salivary cortisol dynamics (CAR, AUCi, AUCg) and sAA levels. (ii) To examine longitudinal associations between AS and eating-related patterns, nutritional and somatic indicators, and depressive symptoms in university students. (iii) To evaluate within-semester changes in AS and their association with trajectories of diet quality, nutritional status, sleep quality, and cardiometabolic risk indicators across the academic semester.

**Study design:** A longitudinal, observational, nonexperimental design with repeated measurements will be implemented across an academic semester. Eating-related patterns, nutritional indicators, depressive symptoms, and stress-related somatic indicators will be assessed repeatedly in the same participants over time, without deliberate manipulation of any variable. The study will be conducted in the students’ natural academic environment.

**Methodology:** A quantitative methodology will be applied, integrating standardized questionnaires, nutritional assessments, biological parameters, wearable-derived records, and salivary indicators. Assessments will be conducted across three time points during the academic semester and will include the following components:

(i) **Psychological and eating-related assessments:** Participants will complete standardized questionnaires assessing academic stress, eating-related patterns, and depressive symptoms. These instruments will be administered during each assessment cycle to capture within-semester variation.
(ii) **Nutritional and cardiometabolic assessments:** Structured nutritional interviews will be conducted to characterize diet, nutritional status, and cardiometabolic risk indicators, including blood pressure. Peripheral blood samples will be collected at baseline and at the end of the semester to determine lipid profile, fasting blood glucose, albumin, globulin, and total protein concentrations.
(iii) **Continuous somatic monitoring:** A low-cost wearable device will be used to record heart rate, oxygen saturation, and sleep-related metrics during the monitoring periods. These outputs will be treated as nonclinical somatic indicators intended to capture within-person patterns over time rather than clinical-grade absolute values. Because the device operates as a closed system and does not allow clinical calibration, we will implement pragmatic data-quality procedures to support longitudinal analyses, including device functionality checks at delivery, monitoring of data completeness, and plausibility rules focused on identifying likely artifacts (e.g., prolonged flatline segments, extended missingness, or physiologically incompatible sequences). Importantly, sleep metrics will not be filtered using rigid assumptions about minimum sleep duration, given that zero recorded sleep may reflect true behavior in some instances, and will instead be evaluated in conjunction with completeness and consistency indicators [78,79].
(iv) **Salivary physiological indicators:** Participants will collect saliva samples twice per week during each assessment cycle. Samples will be analyzed to determine salivary cortisol and salivary alpha-amylase (sAA) levels, following standardized procedures for collection, handling, and storage.

**Universe and sample:** The study population will comprise undergraduate students enrolled in the Faculty of Medicine at the University of Concepción (Chile). A feasibility-based convenience sampling strategy will be used, including students who meet the inclusion criteria and provide informed consent during the recruitment period. Given the longitudinal design with repeated measurements, all eligible participants who consent will be enrolled at baseline. Based on operational capacity and available resources to manage repeated assessments (including biological sampling and wearable monitoring), we will aim to recruit approximately 100–110 participants at baseline. Because follow-up completeness may vary across assessment cycles, the analysis plan will accommodate unbalanced repeated measures.

### Procedure

**Participants:** The study will include undergraduate students enrolled in the Faculty of Medicine at the University of Concepción, excluding first-year students and those completing their professional internships. All participants must sign an informed consent form prior to study enrollment.

**Timing:** Data collection will be organized into three assessment cycles during the academic semester, each lasting three weeks. In each cycle, participants will complete self-administered questionnaires, undergo nutritional interviews, and provide saliva samples according to the protocol schedule. Peripheral blood sampling will be performed at baseline and at the end of the semester to determine biochemical parameters. Somatic indicators will be recorded using wearable devices during the monitoring periods, enabling longitudinal tracking of behavioral, physiological, and psychological variation across the semester. Recruitment is planned to begin in early April 2026, aligned with the start of the academic semester in Chile.

**Recruitment:** Students will be invited through institutional email distributed by the corresponding academic programs and through official study announcements on social media. Participants who accept the invitation will receive standardized study information and instructions, along with the materials required for saliva collection and a wearable device for monitoring.

**Compensation:** Participation is voluntary and no monetary incentive will be provided. Wearable devices will be provided new and unused, and participants will keep the device after completing the monitoring procedures.

**Inclusion criteria:** Eligible participants will be active undergraduate students enrolled in the Faculty of Medicine at the University of Concepción, specifically from the second, third, or fourth year of study, and under 25 years of age at enrollment. This range is intended to maintain comparability in academic structure and developmental stage and to reduce heterogeneity related to markedly different exposure patterns and non-academic responsibilities that may affect sleep, diet, and physiological indicators. All participants must provide written informed consent and complete standardized training for saliva collection procedures and wearable use. Students currently receiving psychological or pharmacological treatment will be eligible to enhance ecological validity and reflect real-world heterogeneity in university populations, where mental health symptoms and service use are common [3,80,81]. Treatment status will be recorded and considered analytically through covariate adjustment and sensitivity analyses.

**Exclusion criteria:** First-year students and students completing professional internships/residencies will be excluded because their academic structure and exposure patterns differ substantially from standard semester-based coursework, which could compromise comparability across assessment cycles. Individuals under 18 years of age will be excluded because participation as a minor would require additional consent procedures (e.g., parental/guardian authorization) and may introduce heterogeneity related to developmental stage and living arrangements that could affect sleep, diet, and stress-related indicators. Additional exclusion criteria will include pregnancy; a self-reported clinical diagnosis of an eating disorder within the previous six months; age 25 years or older; and voluntary withdrawal at any time during the study.

The instruments used for data collection are summarized in Table 1.

**Table 1:** Study variables and measurement instruments.

Domain	Construct/variable	Instrument/source	Key outputs (examples)	Assessment schedule	Methodological notes
Main exposure	Academic stress (AS)	SISCO-II Academic Stress Inventory	Total score; stressors; reactions; coping; reaction subdomains	Each assessment cycle (Cycle 1, Cycle 2, Cycle 3)	Interpretation based on Chilean norms as specified in the protocol
Complementary exposure	Perceived stress	PSS-14	Total score	Each assessment cycle (Cycle 1, Cycle 2, Cycle 3)	Internal consistency will be reported in the study sample
Physiological indicators	HPA-related activity	Salivary cortisol (ELISA)	CAR; AUCi; AUCg (post-awakening window)	Twice per week during each assessment cycle; 3 samples per day (0, + 30, + 60 min)	Sampling compliance criteria specified in Methods
	Sympathetic arousal	Salivary alpha-amylase (sAA activity)	sAA activity (per protocol)	Same samples as cortisol	Noninvasive indicator; standardized spectrophotometric method
Eating-related patterns	Addictive-type eating pattern	YFAS 2.0	Symptom-based scoring outputs (per manual)	Each assessment cycle (Cycle 1, Cycle 2, Cycle 3)	Screening instrument, not a clinical diagnosis
	Grazing / repetitive eating	Grazing Questionnaire	Total score; factors	Each assessment cycle (Cycle 1, Cycle 2, Cycle 3)	Pattern characterization
Diet	Dietary intake	24-hour dietary recall (multi-pass)	Energy; macro- and micronutrients; food-group estimates	Three non-consecutive 24 hour recalls per cycle (Cycle 1, Cycle 2, Cycle 3)	Each 24-hour recall will be treated as a time-specific observation and modeled longitudinally as a time-varying measure. Harmonized collection and coding procedures and a single food composition database will be used across cycles to ensure comparability.
	Dietary inflammatory potential	DII	DII score	Derived from each 24-hour dietary recall (three per cycle) across Cycles 1–3	Standard computation method: calculated at the recall level and modeled as a time-varying measure using harmonized coding across cycles.
	Diet quality (Chile)	IDM-Chile (self-administered)	Total score; categories (high/moderate/low adherence)	Each assessment cycle (Cycle 1, Cycle 2, Cycle 3)	Independent diet-quality indicator
Nutritional status	Anthropometry	Weight, height, waist circumference	BMI; waist circumference (central adiposity)	Baseline and end of semester	Standardized procedures
Cardiometabolic risk	Blood pressure	Standard BP measurement	SBP/DBP	Baseline and end of semester	Coordinated with biomarker visit(s)
	Clinical biochemistry	Peripheral blood sampling	Total cholesterol, LDL-C, HDL-C, triglycerides; fasting glucose; albumin, globulin, total proteins	Baseline and end of semester	Laboratory procedures as specified in Methods
	Metabolic syndrome	Prespecified criteria	Presence/absence; component criteria	Baseline and end of semester	Derived from waist, BP, triglycerides, HDL-C, glucose (and medication when applicable)
Somatic indicators (wearable)	Sleep-related patterns	Redmi Watch 5 Active	Sleep duration; sleep efficiency; device-estimated sleep stages (e.g., REM/non-REM, as provided)	Monitoring periods during each assessment cycle	Nonclinical indicators; QC and completeness monitoring per Methods
	Cardiovascular-related metrics	Redmi Watch 5 Active	Heart rate; SpO₂	Monitoring periods during each assessment cycle	Derived metrics used; raw time-series signals not reconstructed
Psychological symptoms	Depressive symptoms	PHQ-9	Total score; severity categories	Each assessment cycle (Cycle 1, Cycle 2, Cycle 3)	Internal consistency will be reported in the study sample
Covariates	Demographics and context	Self-report items	Sex; age; year of study (2nd–4th); academic workload; chronic conditions; treatment status; etc.	Baseline and updates per assessment cycle as applicable	Illicit drug use is not assessed and is acknowledged as a limitation

Abbreviations: AS, academic stress; AUCg, area under the curve with respect to ground; AUCi, area under the curve with respect to increase; BMI, body mass index; BP, blood pressure; CAR, cortisol awakening response; DII, Dietary Inflammatory Index; ELISA, enzyme-linked immunosorbent assay; HDL-C, high-density lipoprotein cholesterol; HPA, hypothalamic-pituitary-adrenal; IDM-Chile, Chilean Self-administered Mediterranean Diet Index; LDL-C, low-density lipoprotein cholesterol; PHQ-9, Patient Health Questionnaire-9; PSS-14, Perceived Stress Scale (14 items); REM, rapid eye movement; sAA, salivary alpha-amylase; SBP/DBP, systolic/diastolic blood pressure; SpO₂, peripheral oxygen saturation; TG, triglycerides; YFAS 2.0, Yale Food Addiction Scale 2.0.

### Instruments for assessing academic stress and perceived stress

**SISCO-II academic stress inventory (SISCO-II):** The SISCO-II is a 33-item instrument designed to assess academic stress as a multidimensional construct. It comprises three domains: academic stressors (8 items), reactions (17 items), and coping strategies (6 items). The reactions domain is further differentiated into physical and psychological reactions, and social and behavioral reactions. Items are answered on a five-point Likert scale ranging from 1 (never) to 5 (always). For this study, interpretation will be based on normative data established for Chilean university students, allowing contextualized scoring. In the stressors domain, scores below the 40th percentile are classified as low, between the 40th and 60th percentiles as moderate, and above the 60th percentile as high. In the reactions domain, scores below the 35th percentile indicate low reactivity, between the 35th and 65th percentiles indicate moderate reactivity, and above the 65th percentile indicate high reactivity. Robust psychometric properties and internal consistency for the total scale and its domains have been reported, including evidence derived from Chilean validation and scoring studies, supporting its use in this protocol [29,30,82].

**Perceived stress scale (PSS14):** The PSS-14 is a widely used measure of perceived stress that assesses the extent to which individuals appraise their life situations as stressful [83]. Psychometric evidence supports its use across diverse populations, including European Spanish validation studies and broader reviews of the instrument’s measurement performance [84,85]. In Chilean samples, the PSS has also been applied in research settings, supporting its feasibility of use in the local context [86]. In the present study, internal consistency will be reported for the total score and relevant subscales in the study sample.

### Instruments for assessing nutritional variables (diet and nutritional status)

The instruments used to evaluate dietary intake and nutritional status are described below.

### Diet assessment

**24-hour dietary recall:** Dietary intake will be assessed using a structured, interviewer-administered 24-hour dietary recall. Individual interviews will be conducted by a trained nutritionist on three non-consecutive days during each assessment cycle to estimate total food and beverage intake over the preceding 24 hours. To ensure methodological consistency across cycles, dietary recalls will be standardized through uniform collection, evaluation, and coding procedures applied throughout the study. Each recall will be treated as a time-specific observation and modeled longitudinally as a time-varying measure, without averaging across cycles. Comparability across measurement occasions will be supported by identical assessment procedures, standardized coding criteria, and a single food composition database to estimate total energy intake and macro- and micronutrient composition [87]. A standardized multi-pass approach will be used to reduce recall bias and improve completeness [88].

**Dietary Inflammatory Index (DII):** The DII is a literature-derived index designed to estimate the overall inflammatory potential of the diet along a continuum from more anti-inflammatory to more pro-inflammatory [89]. In this protocol, the DII will be computed using dietary intake data derived from the 24HR assessments, following the standard method described by the developers [89]. The index is supported by evidence linking specific dietary components to systemic inflammatory markers and is applicable across populations where dietary intake data are available [90].

**Chilean Self-administered Mediterranean Diet Index (IDM-Chile):** The IDM-Chile is a self-administered instrument developed to assess adherence to the Mediterranean dietary pattern in the Chilean population as an indicator of diet quality [91]. It includes 14 items assessing portion size and frequency of consumption across key food groups. Items are scored from 0 to 2 according to predefined criteria, yielding a total score that is classified as high adherence (9–14 points), moderate adherence (5–8.5 points), or low adherence (<5 points) [91]. High reliability has been reported in a large Chilean adult sample (Spearman’s ρ = 0.94) [91].

### Assessment of nutritional status

**Anthropometric measurements:** Weight, height, and waist circumference will be assessed as indicators of nutritional status and body composition. Weight and height will be used to calculate body mass index (BMI), expressed as kg/m², and categorized according to World Health Organization criteria [92]. Waist circumference will be used as an indicator of central adiposity, applying cut-off points of ≥80 cm for women and ≥90 cm for men [93]. Anthropometric measurements will be obtained using standardized procedures following established guidelines for nutritional assessment [94].

**Biochemical parameters associated with nutritional status:** Metabolic and protein-related parameters will be assessed using peripheral blood samples, including fasting blood glucose, lipid profile, albumin, globulin, and total protein concentrations. Analyses will be performed using standardized automated spectrophotometric procedures routinely used in clinical chemistry laboratories, following laboratory quality procedures and manufacturer instructions [95].

### Instruments for assessing eating behavior

**Yale Food Addiction Scale 2.0 (YFAS-2.0):** The YFAS 2.0 is used as a screening instrument to identify addictive-type eating patterns based on the 11 substance use disorder criteria described in the DSM-5 framework. It includes 35 items rated on a Likert-type frequency scale ranging from 0 (never) to 7 (every day), allowing estimation of symptom presence and related scoring outputs according to the instrument guidelines [46]. The YFAS 2.0 has been validated in Spanish and has shown good psychometric performance [96,97]. In Chile, validation evidence in nonclinical samples, including university students, has reported high reliability (McDonald’s ω = 0.88), supporting its use in this protocol [98].

**The grazing questionnaire:** The Grazing Questionnaire assesses repetitive, often unplanned consumption of small amounts of food over extended periods. It comprises seven items grouped into two factors. The instrument has shown good internal consistency (Cronbach’s α = 0.82) in university samples without a clinical history [43]. In this protocol, the questionnaire will be applied to characterize grazing-related patterns in the study population.

### Instruments for assessing somatic variables (sleep and cardiovascular risk factors)

**Sleep patterns:** Sleep-related patterns will be assessed using a wearable device (Redmi Watch 5 Active), which provides device-derived sleep metrics during the monitoring periods. Outputs will include sleep duration, sleep efficiency, and device-estimated sleep stages (e.g., REM and non-REM stage estimates, as defined by the device). These outputs will be treated as nonclinical somatic indicators intended to capture within-person patterns over time rather than clinical-grade measurements [78,79].

**Cardiovascular risk factors in young adults:** Cardiometabolic risk will be examined across three complementary domains: (i) behavioral risk factors (e.g., smoking, alcohol use, physical inactivity, and sleep-related problems) [71]; (ii) biochemical and metabolic indicators derived from blood sampling (e.g., lipid profile and fasting glucose) [72]; and (iii) metabolic syndrome, operationalized using standard criteria defined a priori in the protocol.

**Metabolic syndrome (operational definition):** Metabolic syndrome will be defined using the harmonized criteria (Joint Interim Statement), which considers the presence of any three of five components: elevated waist circumference (using country- or ethnicity-specific cutoffs), elevated triglycerides, reduced HDL-cholesterol, elevated blood pressure, and elevated fasting glucose [99]. In this protocol, waist circumference cutoffs will follow the thresholds already specified for central adiposity (≥80 cm for women and ≥90 cm for men), and medication for any component will be treated as meeting the corresponding criterion when applicable.

### Instrument for assessing depressive symptoms

**Patient Health Questionnaire-9 (PHQ-9, Spanish version):** Depressive symptoms will be assessed using the PHQ-9, a self-administered measure based on DSM symptom criteria for major depressive disorder [100]. The instrument consists of nine items with Likert-type response options and has shown good reliability in Chilean samples [101].

**Data collection mechanism:** Data collection and management will be conducted using REDCap (Research Electronic Data Capture), a secure, web-based platform designed for biomedical and academic research [102]. The platform will be used to administer online surveys, store data centrally, and support confidentiality and access control. Participants will receive survey links via text message, allowing completion from mobile phones or computers without requiring additional applications. REDCap will also support automated reminders and real-time monitoring of data collection progress.

**Use of smart devices:** The study will incorporate smart wristband-type devices selected for affordability, broad availability, and feasibility for implementation in university populations. These devices enable non-invasive recording of somatic indicators such as heart rate, oxygen saturation, and sleep-related metrics during monitoring periods [78,79]. Because the device operates as a closed system and does not allow clinical calibration, wearable outputs will be treated as nonclinical indicators and used to identify longitudinal patterns rather than clinical-grade absolute values. Standardized procedures will be applied to support data quality and device functioning, including device delivery checks, monitoring of completeness, and artifact-oriented plausibility rules (e.g., extended missingness or prolonged flatline segments). No rigid assumptions about minimum sleep duration will be used, because extremely short or even zero recorded sleep may reflect true behavior in some instances. Wearable data completeness will be monitored throughout the monitoring periods. Missingness will be handled at the analysis stage using the prespecified statistical strategy, based on derived wearable metrics rather than imputing raw time-series signals.

### Collection of saliva samples

Saliva samples will be collected twice per week during each assessment cycle to quantify salivary cortisol and sAA as noninvasive physiological indicators of stress-related activation [11,12,15]. Before starting, participants will receive practical training and written instructions to support standardized home collection using Salivette® devices.

**Sampling schedule and compliance procedures:** Each sampling day will include three consecutive samples: immediately upon awakening (0 min), and 30 and 60 minutes thereafter. **Sampling compliance criteria:** To compute CAR and AUC-derived indices, the awakening sample will be considered compliant when collected within 5 minutes of self-reported wake time. The + 30 and +60 minute samples will be considered compliant when collected within ±10 minutes of their target times. Sampling days outside these windows will be flagged and excluded from CAR/AUC computation and considered in sensitivity analyses [20]. Participants will record wake time and sampling times using time-stamped entries in REDCap and will be instructed to follow standardized pre-collection conditions (e.g., no food or beverages except water, no smoking, and no tooth brushing immediately before collection). Because CAR estimates are sensitive to deviations from the awakening schedule, time compliance will be monitored, and noncompliant sampling days will be flagged for sensitivity analyses [20]. Wearable-derived sleep timing will be used as an auxiliary reference to support consistency checks of reported wake time when available.

**Handling, labeling, and storage:** Samples will be labeled with unique participant codes to ensure traceability while preserving confidentiality. Participants will store samples under refrigeration until delivery to the research team according to study instructions. In the laboratory, samples will be processed following standardized procedures, including centrifugation, and stored at −20 °C until analysis to preserve sample integrity.

### Determination of salivary cortisol

Frozen samples will be thawed under controlled conditions prior to analysis. Cortisol concentration will be determined using an ELISA-based immunoassay with the commercial Salivary Cortisol ELISA Kit (Eagle Biosciences, CRT32-K01), following the manufacturer’s instructions [103]. Absorbance will be read at a primary wavelength of 405 nm with spectral correction between 570 and 590 nm, as specified by the kit insert. Cortisol awakening response (CAR) metrics will be derived from the serial post-awakening measurements, and the AUCi and AUCg indices will be computed to characterize cortisol dynamics within the post-awakening sampling window [20].

**Determination of salivary alpha-amylase:** Salivary alpha-amylase activity will be assessed using a standardized spectrophotometric method based on 2-chloro-p-nitrophenyl-maltotriose (CNPG₃) as substrate. Hydrolysis releases 2-chloro-4-nitrophenol, which will be quantified at 405 nm, with the rate of increase in absorbance proportional to enzymatic activity. The procedure will follow the laboratory protocol and reagent specifications for clinical chemistry photometric methods [95,104], and sAA will be interpreted as a noninvasive indicator of sympathetic arousal in psychophysiological research [13–15].

**Blood sample collection:** Peripheral venous blood samples will be obtained at the Neuroscience, Psychiatry, and Mental Health Laboratory of the University of Concepción (NEPSAM-UdeC). Blood draws will be performed by a Medical Technologist trained in phlebotomy and biospecimen handling procedures. Sample collection will be scheduled to coincide with anthropometric assessment, blood pressure measurement, and the dietary interview to optimize integration of physiological and behavioral data. Participants will be instructed to arrive after at least 12 hours of fasting.

### Determination of biochemical parameters

Biochemical determinations will include lipid profile (total cholesterol, LDL-cholesterol, HDL-cholesterol, and triglycerides), fasting blood glucose, albumin, globulin, and total proteins as metabolic and protein-related indicators of nutritional status. Analyses will be conducted using standardized automated spectrophotometric procedures with commercial reagents from Human Diagnostics Worldwide [104]. After collection, tubes will be centrifuged at 2500 × g for 10 minutes. Serum or plasma will be separated according to the analytical requirements of each parameter and stored in labeled microtubes at −80 °C until analysis.

### Statistical analysis

Statistical analyses will be conducted in R [105]. Analyses will be performed in three main stages:

(i) **Descriptive analysis:** Sample characteristics will be summarized using measures of central tendency and dispersion (mean and standard deviation for approximately normally distributed variables; median and interquartile range for skewed variables). Categorical variables will be described using absolute frequencies and percentages. Distributions will be inspected graphically and, when appropriate, transformations will be considered for markedly skewed biomarkers.
(ii) **Bivariate analysis:** Associations between variables will be explored using graphical inspection and correlation analyses. Correlations will be computed using Pearson or Spearman coefficients depending on distributional assumptions and will be examined at each assessment cycle to describe contemporaneous relationships. Correlation matrices will be used to identify patterns among key variables, including AS, eating-related patterns, diet indices, nutritional status indicators, sleep-related metrics, cardiometabolic indicators, and depressive symptoms.
(iii) **Multivariate and longitudinal analysis:** To examine adjusted associations, regression models will be specified according to outcome type (linear models for continuous outcomes; generalized models, including logistic models, for categorical outcomes). To evaluate within-participant changes over time, mixed-effects longitudinal models will be used, including participant-level random intercepts to account for within-person correlation across repeated measurements. Time will be represented by assessment cycle, and key predictors (including AS and relevant covariates) will be treated as time-varying when applicable. Dietary variables derived from the 24-hour dietary recalls (including DII-derived measures) will be analyzed at the recall level as time-specific observations, nested within assessment cycles, and modeled longitudinally as time-varying outcomes within mixed-effects models, without averaging across cycles. Wearable data will be analyzed using derived metrics (e.g., daily or cycle-level summaries) rather than raw time-series signals. Illicit drug use was not assessed in this protocol and will therefore be considered as a potential source of residual confounding, particularly for outcomes related to sleep and mood.
(iv) RI-CLPM analyses will be restricted to constructs assessed once per assessment cycle; therefore, diet in the cross-lagged framework will be represented using the cycle-level IDM-Chile. To examine temporal cross-lagged associations while separating stable between-person differences from within-person fluctuations, the random-intercept cross-lagged panel model (RI-CLPM) will be applied across the three assessment cycles. This approach will be used to evaluate cross-lagged associations between AS and selected outcomes (e.g., depressive symptoms, eating-related patterns, diet indices, sleep-related metrics, and cardiometabolic indicators) and to explore reciprocal patterns where theoretically justified. All interpretations will be framed as associational given the observational design.

**Missing data:** Because incomplete follow-up and intermittent missingness are expected in repeated-measures designs, missing data will be addressed using the prespecified longitudinal strategy. Mixed-effects models will be estimated using likelihood-based approaches that accommodate unbalanced repeated measures under Missing At Random assumptions. For RI-CLPM and related longitudinal models, full-information estimation will be used when applicable. If required (e.g., missing covariates), multiple imputation procedures will be implemented at the level of derived variables, and sensitivity analyses will be conducted to assess robustness to missingness patterns.

**General data plan:** At the end of the project, all de-identified data generated will be shared with the scientific community through deposition in an open-access repository. The dataset will be deposited in the University of Concepción institutional Dataverse repository (Dataverse UdeC), together with a data dictionary/codebook and sufficient metadata to support transparency, independent verification, and reproducibility. Procedures for data organization, secure storage, access control, and confidentiality will be implemented throughout the study.

**Publication and dissemination of protocol results:** Before any public dissemination, all study data will undergo a structured de-identification process to remove direct identifiers and reduce re-identification risk. Once de-identified, the dataset, together with a codebook and relevant metadata, will be deposited in the University of Concepción institutional repository (Dataverse UdeC) to support transparency, independent verification, and reproducibility. If any subset of variables is considered potentially identifying, access restrictions will be applied according to repository policies, while maintaining public metadata describing the dataset.

## Discussion

University settings expose students to sustained academic demands, evaluative pressure, and the daily work of adapting to new social and personal contexts. In that landscape, AS becomes more than a transient discomfort. When it is persistent, it may co-occur with changes in sleep, mood, eating-related patterns, and cardiometabolic risk indicators. Yet, despite the frequency with which these links are mentioned, the mechanisms and temporal dynamics that connect AS to such outcomes remain only partially understood, especially when AS is not operationalized consistently and when biological indicators are not incorporated.

A methodological limitation is difficult to ignore. Much of the available literature is cross-sectional and largely self-report based, which restricts what can be concluded about within-person change across the semester and about the ordering of events over time. What this protocol offers is a repeated-measures framework that brings physiological indicators (salivary cortisol dynamics and sAA), wearable-derived nonclinical somatic indicators, and validated questionnaires into the same longitudinal model. The goal is not to replace subjective experience with devices, but to connect self-reported stress to concurrent and time-varying physiological and behavioral patterns. In doing so, the protocol provides a structured basis for testing hypotheses with greater temporal resolution and for informing prevention and early intervention strategies that are better aligned with the lived reality of university students.

Young university students may be particularly vulnerable to AS, which has been associated with both mental health symptoms and early indicators of physical health risk. Reports in student populations describe high levels of anxiety and depressive symptoms alongside increasing rates of excess body weight and early cardiometabolic risk profiles [3,81,106,107]. This combination suggests a dual psychological and metabolic burden that can be amplified during periods of sustained academic demand.

Sustained stress-related activation may co-occur with changes in sleep, eating-related patterns, and emotional well-being, which are domains directly captured by the present protocol [9,63,64]. In this context, stress-related eating patterns and poorer sleep may align with less favorable cardiometabolic profiles over time, supporting the rationale for integrating behavioral measures, physiological indicators, and cardiometabolic outcomes within the same longitudinal framework [54,68].

Chronic stress has been associated with a higher risk of developing and maintaining depressive symptoms, plausibly through disruptions in emotional regulation and stress-related biological pathways, including HPA-axis dysregulation and inflammatory processes [10,73,77,108]. In this protocol, depressive symptoms are therefore included as a key psychological outcome to be examined longitudinally alongside AS and physiological indicators.

A methodological strength of this protocol is the integration of complementary stress-related indicators. sAA is incorporated as a noninvasive indicator of autonomic arousal, which has shown sensitivity to psychosocial stress contexts and can be feasibly implemented in repeated-measures designs [13–15]. Combined with salivary cortisol as an HPA-related indicator, this approach supports a more complete characterization of stress-related activation across the semester. To retain the subjective and contextual dimension of the construct, AS is operationalized using the SISCO-II framework, which allows interpretation based on Chilean validation and normative evidence and supports identification of subgroups with different stress profiles [29,30].

A further strength of this protocol is the integration of dietary assessment with complementary diet-quality and inflammatory-potential indices. Dietary intake will be captured using repeated 24-hour dietary recalls, a widely used method in nutritional epidemiology that benefits from a structured multi-pass approach [88,109]. By combining repeated 24-hour dietary recalls and recall-level DII measures with the cycle-level IDM-Chile, the protocol enables a joint examination of diet quality and dietary inflammatory potential alongside AS and physiological outcomes, without assuming causal pathways in advance [89,91]. In parallel, the integration of standardized anthropometry with biochemical parameters supports longitudinal description of cardiometabolic risk-related profiles in the study population.

Regarding eating-related patterns, the combined use of the YFAS 2.0 and the Grazing Questionnaire supports characterization of complementary, conceptually distinct dimensions. The YFAS 2.0 captures addictive-type eating patterns using a symptom-based framework, whereas the Grazing Questionnaire focuses on repetitive, often unplanned intake of small amounts of food that may occur outside discrete binge episodes [43,46]. Together, these instruments allow differentiation between loss-of-control–type patterns and habitual grazing behaviors, enabling longitudinal examination of how within-semester variation in AS aligns with different eating-related profiles across assessment cycles.

The PHQ-9 provides a standardized operationalization of depressive symptoms and supports comparability with prior work in student and primary care populations [100,101]. Its repeated administration across assessment cycles allows depressive symptom variation to be examined alongside AS and the protocol’s physiological indicators within the longitudinal framework.

Overall, the protocol’s main contribution is methodological: it integrates physiological indicators, wearable-derived somatic metrics, and validated self-report measures within a repeated-measures framework across an academic semester. This structure supports a coherent description of within-person variability and co-occurrence patterns across behavioral, somatic, and psychological domains in relation to AS, providing a practical template for future longitudinal research in university settings.

## Limitations

This protocol has several limitations that should be considered when interpreting future results. First, some key constructs rely on self-report (e.g., perceived stress, depressive symptoms, eating-related patterns, and parts of diet assessment), which may introduce reporting and recall biases despite standardized administration procedures.

Second, the sample is feasibility-based and restricted to undergraduate students from a single Faculty of Medicine and a single institution, within a defined age range and academic stage (2nd–4th year). These design choices support operational consistency across assessment cycles, but they limit external validity and the transferability of findings to other universities, regions, and student profiles.

Third, wearable-derived outputs are treated as nonclinical somatic indicators. The selected device is a closed system and does not allow clinical calibration; therefore, measurement error is expected, particularly for sleep-stage estimates. The protocol addresses this by focusing on within-person patterns and derived metrics rather than clinical-grade absolute values [78,79].

Fourth, repeated biological sampling depends on participant adherence. Although strict compliance criteria are used for post-awakening saliva collection, deviations can occur and may reduce usable observations for CAR-derived indices. In addition, illicit drug use was not assessed; residual confounding cannot be ruled out, particularly for sleep and mood outcomes.

Finally, including participants undergoing psychological or pharmacological treatment may increase heterogeneity in physiological and psychological measures. This decision reflects the real student context and supports ecological validity, but treatment status will need to be handled analytically as a potential confounder. A further limitation is the potential overrepresentation of women, given known sex differences in stress reactivity and symptom expression [3,80,81].

Despite these limitations, the protocol provides a replicable framework for integrating repeated physiological indicators, wearable-derived metrics, and validated self-reports across an academic semester. This structure can be extended in future studies with larger and more diverse samples.

### Strengths

A main strength of this protocol is the repeated-measures, longitudinal design across an academic semester. This structure supports the examination of within-person variability over time and reduces reliance on cross-sectional snapshots, allowing a more precise characterization of how AS aligns with concurrent changes across physiological, behavioral, somatic, and psychological domains.

A second strength is the feasibility-oriented integration of multiple measurement modalities using scalable procedures. The protocol combines noninvasive salivary indicators with wearable-derived nonclinical somatic metrics and validated self-report instruments, creating a replicable framework that can be implemented in university settings. This integrative approach provides a practical methodological template for future longitudinal and comparative research on AS.

## Conclusion

This protocol outlines a multidimensional, repeated-measures approach to examining AS by integrating physiological indicators, eating-related patterns, dietary and nutritional measures, somatic indicators, and depressive symptoms in university students across an academic semester. By combining scalable, noninvasive procedures with validated self-report instruments, the study is intended to generate longitudinal evidence on how AS co-occurs with changes across these domains. The framework may support the development of prevention-focused strategies and inform university-level health promotion efforts, while providing a replicable template for future research in comparable student populations.
